# Effects of Nutritional Strategies on Glucose Homeostasis in Gestational Diabetes Mellitus: A Systematic Review and Network Meta-Analysis

**DOI:** 10.1155/2020/6062478

**Published:** 2020-02-23

**Authors:** Shixiao Jin, Liyan Sha, Jianli Dong, Jing Yi, Yang Liu, Zhongxian Guo, Bing Hu

**Affiliations:** ^1^Department of Nursing, The Second Affiliated Hospital of Dalian Medical University, Dalian 116000, China; ^2^Department of International Medicine, The Second Affiliated Hospital of Dalian Medical University, Dalian 116000, China; ^3^Department of Gynaecology and Obstetrics, The Second Affiliated Hospital of Dalian Medical University, Dalian 116000, China

## Abstract

**Background:**

Gestational diabetes mellitus (GDM) is one of the most common complications of pregnancy, and nutritional therapy is the basis of GDM treatment. However, the effects of different forms of nutritional supplementation on improving gestational diabetes are uncertain.

**Objective:**

We conducted a network meta-analysis to evaluate the effects of supplementation with different nutrients on glucose metabolism in women with GDM.

**Methods:**

We conducted a literature search using PubMed, EMBASE, and the Cochrane Library to identify randomized controlled trials (RCTs) comparing the differences between different nutritional strategies in women with GDM. The Cochrane tool was used to assess the risk of bias. Pairwise meta-analysis and network meta-analysis were used to compare and rank the effects of nutritional strategies for the improvement of fasting plasma glucose (FPG), serum insulin, and homeostasis model assessment-insulin resistance (HOMA-IR).

**Results:**

We included thirteen RCTs with a total of 754 participants. Compared with placebo, omega-3, magnesium, vitamin D, zinc, and probiotics were more beneficial for improving FPG, serum insulin, and HOMA-IR. Network analysis showed that vitamin D supplementation was superior to omega-3 (-3.64 mg/dL, 95% CI: -5.77 to -1.51), zinc (-5.71 mg/dL, 95% CI: -10.19 to -1.23), probiotics (-6.76 mg/dL, 95% CI: -10.02 to -3.50), and placebo (-12.13 mg/dL, 95% CI: -14.55 to -9.70) for improving FPG. Magnesium supplementation was more beneficial for decreasing serum insulin compared with probiotics (-5.10 *μ*IU/mL, 95% CI: -9.32 to -0.88) and placebo (-7.80 *μ*IU/mL, 95% CI: -9.32 to -0.88) and placebo (-7.80

**Conclusion:**

Vitamin D supplementation significantly reduced FPG and regulated HOMA-IR. Magnesium supplementation was superior in decreasing serum insulin than supplementation with other nutrients. Nutrient supplementation seemed to have an effect on glucose homeostasis maintenance in patients with GDM and may be considered an adjunctive therapy.

## 1. Introduction

Gestational diabetes mellitus (GDM) is defined as any degree of glucose intolerance with an onset or first recognition during pregnancy [1] and is one of the most common complications during pregnancy. GDM affects 9.3%-25.5% of pregnant women, and its incidence is continuing to increase [2]. GDM is considered to be a form of impaired glucose tolerance similar to prediabetes in nonpregnant individuals and has become a global public health problem that is associated with short-term and long-term adverse health problems in mothers and their offspring [3]. Severe GDM increases the risk of spontaneous abortion and preeclampsia during pregnancy and can lead to the occurrence of congenital abnormalities, fetal macrosomia, and hypoglycemia in newborns [4, 5]. Studies have shown that GDM is a major risk factor for the development of impaired glucose tolerance and type 2 diabetes mellitus among pregnant women; women develop diabetes mellitus at rates of 20–60% within 5–10 years after pregnancy [6, 7]. The risks of developing metabolic syndrome and cardiovascular disease are three times higher in women with GDM. In addition, children born to women with GDM have increased risks of impaired glucose tolerance and obesity.

GDM is thought to be related to the progression of pregnancy; islet resistance is affected by hormones, leading to decreased insulin sensitivity and glucose intolerance [8]. Therefore, it is essential to identify reasonable and effective methods for improving insulin sensitivity and maintaining blood glucose homeostasis. Dietary therapy is considered to be the first-line treatment for GDM [9]. The purpose is to strictly control the glucose levels of pregnant women on the basis of conventional treatment, ensure the reasonable nutritional intake of pregnant women and fetuses, and reduce the occurrence of complications in pregnant women and their children. The International Federation of Obstetrics and Gynecology (FIGO), the American Diabetes Association (ADA), the Canadian Diabetes Association (CDA), and the Japanese Diabetes Association (JDS) expounded the importance of diet for the treatment of gestational diabetes [10–13]. Strictly controlled blood glucose during pregnancy has been shown to reduce the risk of complications; however, at present, no clear guideline for GDM dietary management exists. The dietary advice for GDM patients has focused on limiting carbohydrates [14], and there is still no consensus on the best nutritional strategies for improving blood glucose. The challenge is now to determine which nutritional strategies are reasonable and effective for GDM. Therefore, this study is aimed at evaluating the effectiveness of different nutritional strategies on blood glucose homeostasis in GDM patients and ranking the effectiveness of providing safe and effective methods for the management of GDM.

## 2. Materials and Methods

The study was conducted according to the Preferred Reporting Items for Systematic Reviews and Meta-Analyses (PRISMA) extension statement for reporting network meta-analyses of health care interventions [15].

### 2.1. Search Strategy

The PubMed, EMBASE, and Cochrane Library electronic databases were searched for randomized controlled trials (RCTs) that evaluated GDM and nutritional strategies published between database inception and July 2019 using the following terms: (‘diabetes, gestational' or ‘pregnancy-induced diabetes' or ‘gestational diabetes mellitus' or ‘GDM') AND (‘nutrient^∗^' or ‘nutrition' or ‘dietary supplement' or ‘protein' or ‘amino acids' or ‘fatty acids' or ‘vitamin' or ‘mineral' or ‘antioxidant' or ‘phytochemical') AND (‘randomized controlled trial' or ‘controlled trial' or ‘clinical trial' or ‘random^∗^' or ‘RCT'). We also scanned the reference lists of the retrieved articles to identify additional eligible studies. [Supplementary-material supplementary-material-1] of the supplemental material details the search strategy.

### 2.2. Eligibility Criteria

The eligibility criteria are detailed below following the participants, intervention, controls, outcomes and study design (PICOS) framework [16]: (i) Participants: we included studies enrolling participants with GDM. (ii) Interventions: any RCTs evaluating nutrient supplementation compared with placebo on the basis of receiving nutritional treatment or maintaining the usual diets were included. (iii) Controls: groups receiving placebo or those receiving any nutrient supplementation on the basis of receiving nutritional treatment or maintaining the usual diet were considered. Studies without control conditions were excluded. (iv) Outcomes: the outcome measures were changes in fasting plasma glucose (FPG), serum insulin, and homeostasis model assessment-insulin resistance (HOMA-IR). (v) Studies: only RCTs were considered.

### 2.3. Data Extraction

All retrieved articles were combined in EndnoteX7 to remove duplicates. Two researchers independently screened the titles and abstracts according to the prespecified criteria. The full texts of articles that potentially met the eligibility criteria were reviewed. In cases of disagreement, a third researcher was consulted for a final decision. The two researchers independently extracted and cross-checked the data. For each included study, the following data were extracted: general information, study characteristics, interventions, and outcomes.

### 2.4. Quality Assessment

Cochrane's risk of bias tool was used by two researchers to independently assess the risk of bias [17], including the following: random sequence generation, allocation concealment, blinding of participants and personnel, blinding of outcome assessment, incomplete outcome data, selective reporting, and other bias. Each quality assessment was classified as low risk of bias, high risk of bias, or unclear (moderate risk of bias).

### 2.5. Statistical Analysis

We performed a pairwise meta-analysis using the random effects model for every intervention comparison, and the *I*^2^ statistic and *P* values were calculated as a measure of the statistical heterogeneity [18], with *I*^2^ ≥ 50% indicating substantial heterogeneity.

In addition, network meta-analysis (NMA) was conducted to estimate the comparative effects of different types of nutrient supplementation on maintaining glucose homeostasis. The results of the comparative effects are presented as the weighted mean differences (WMDs) and 95% confidence intervals (CIs). We also estimated the ranking probabilities of the intervention effect using surface under the cumulative ranking curve (SUCRA) [19]. The larger the SUCRA value, the better the ranking of the intervention effect. Transitivity is the basis of NMA [20]. The consistency between the direct and indirect evidence was evaluated using inconsistency tests to assess the validity of the transitivity assumption. Publication biases or small sample effects were examined using a comparison-adjusted funnel plot.

Review Manager version 5.3 and Stata version 15 were used to conduct the analyses, and the extracted data were subjected to in-depth analysis [21]. The “metan” package was used for the pairwise meta-analysis, and the “network” package was used to conduct the NMA. Statistical significance was set as a *P* value < 0.05 in all analyses.

## 3. Results

### 3.1. Study Selection and Characteristics

**Figure 1:**
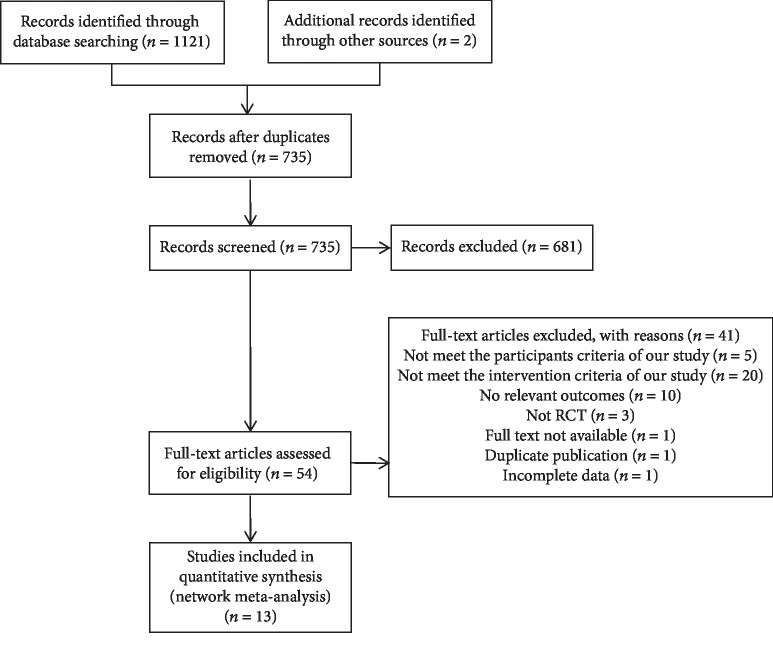
Study flow diagram.

A total of 1121 articles were retrieved by following the predesigned literature retrieval strategy. By further searching the references included in the articles, 2 additional articles were obtained. After reading the titles and abstracts, 54 studies were selected for further review. Finally, thirteen studies met our inclusion criteria [22–34]. The detailed process of the search strategy is described in Figure 1.  Table 1 summarizes the basic characteristics of the included studies. The included studies were published between 2013 and 2019, and eleven trials were from Iran, while the other two were from China and Thailand. A total of 754 participants were included in this review and the average age of the participants was 29.8 years. The mean duration of the intervention was 6.8 weeks. Five studies including 310 participants mentioned mean gestational age, and the mean gestational age of these participants was 22.4 weeks.

### 3.2. Results of Risk of Bias

**Figure 2:**
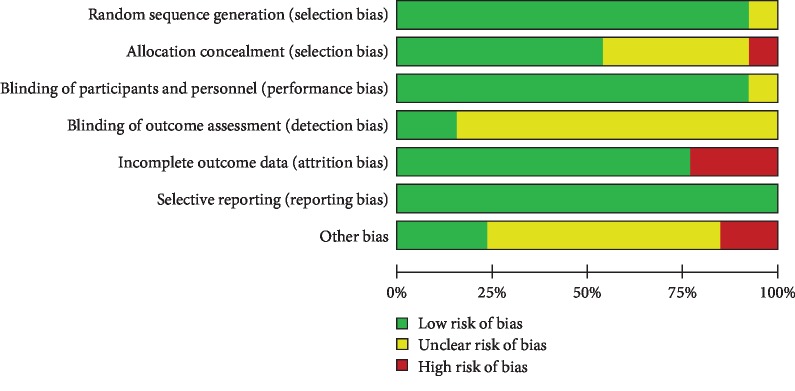
Risk of bias in the included studies.

Figure 2 shows the risk of bias of the included studies. Twelve studies [22–29, 31–34] reported methods for generating random sequences, which were mainly computerized randomization methods. Seven studies mentioned the use of allocation concealment, and three of these studies described specific methods [25, 27, 29]. Five studies [22, 25, 28, 31, 33] clearly illustrated the blinding of participants and personnel. Two studies [25, 27] indicated that the outcome assessors were blinded. Six studies [23–25, 27, 29, 33] clarified that the analytical method was based on intention-to-treat analysis.

### 3.3. Pairwise Meta-Analysis

We used paired meta-analysis to analyze the effects of nutritional interventions on glucose homeostasis in GDM women from three aspects, including FPG, insulin, and HOMA-IR. The results showed that nutritional supplementation was effective in regulating FPG, insulin, and HOMA-IR compared with a placebo. Five comparisons (placebo vs. omega-3, placebo vs. magnesium, placebo vs. vitamin D, and placebo vs. zinc in regulating FPG and placebo vs. probiotics in regulating insulin) showed no heterogeneity. Regarding the regulation of insulin and HOMA-IR, the comparison of placebo vs. zinc showed high heterogeneity (*I*^2^ = 62% and *I*^2^ = 81%, respectively).  Table 2 displays the detailed results. In terms of the source of heterogeneity, the meta-analysis included only two studies, and the study by Roshanravan et al. [30], which did not mention information on allocation concealment or the blinding of the outcome assessment, may have led to higher heterogeneity in the study.

### 3.4. Network Plots

**Figure 3:**
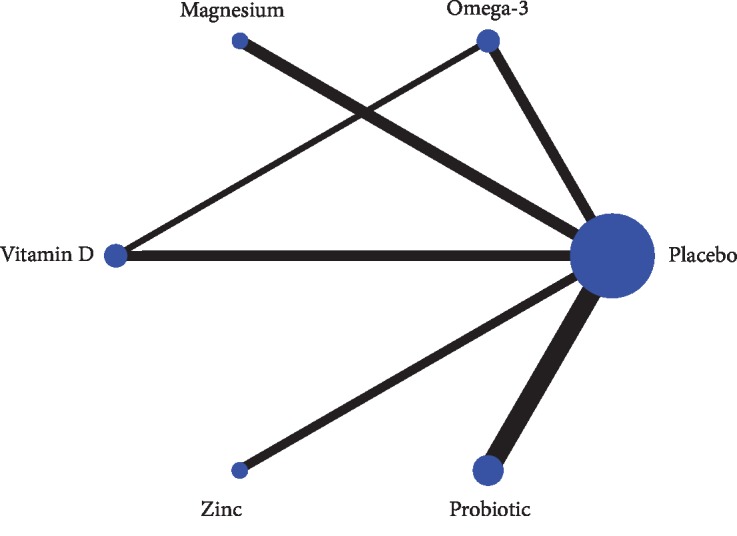
Network plots of eligible comparisons for different nutritional strategies.

Figure 3 shows the network plots of the included studies. We included six interventions in the network meta-analysis: omega-3, magnesium, vitamin D, zinc, probiotic, and placebo. Each point represents an intervention, and the size of the point represents the sample size of the intervention. Lines between points represent the direct comparison evidence, and the number of studies is reflected by the thickness of the line.

### 3.5. Results of Network Meta-Analysis

The inconsistency test showed that FPG (*χ*^2^ = 2.24; *P* = 0.1341), insulin (*χ*^2^ = 0.81; *P* = 0.3678), and HOMA-IR (*χ*^2^ = 056; *P* = 0.4532) exhibited no inconsistencies in the global analysis at the levels of *P* value > 0.05, indicating that the direct comparison and indirect comparison results were consistent.

#### 3.5.1. FPG

Table 3 shows the results of the effects of the interventions on FPG. Thirteen studies reported the impact of different interventions on FPG control. Compared with placebo, vitamin D (-12.13 mg/dL, 95% CI: -14.55 to -9.70), magnesium (-10.59 mg/dL, 95% CI: -13.68 to -7.50), omega-3 (-8.49 mg/dL, 95% CI: -11.28 to -5.70), zinc (-6.42 mg/dL, 95% CI: -10.18 to -2.65), and probiotics (-5.37 mg/dL, 95% CI: -7.54 to -3.19) resulted in a significant reduction in FPG. Compared to probiotics, vitamin D (-6.76 mg/dL, 95% CI: -10.02 to -3.50) and magnesium (-5.22 mg/dL, 95% CI: -9.00 to -1.44) resulted in a greater reduction in FPG. Compared to omega-3 and zinc, vitamin D (-3.64 mg/dL, 95% CI: -5.77 to -1.51; -5.71 mg/dL, 95% CI: -10.19 to -1.23) was more effective in reducing FPG. There were no significant differences between the other interventions in terms of the effectiveness in reducing FPG.

#### 3.5.2. Insulin

Table 4 shows the results of the effects of the interventions on insulin. Eleven studies on insulin regulation were included in our review. Compared to placebo, magnesium (-7.80 *μ*IU/mL, 95% CI: -11.95 to -3.65), vitamin D (-5.89 *μ*IU/mL, 95% CI: -7.57 to -4.22), zinc (-4.78 *μ*IU/mL, 95% CI: -8.16 to -1.41), omega-3 (-4.12 *μ*IU/mL, 95% CI: -6.38 to -1.86), and probiotics (-2.70 *μ*IU/mL, 95% CI: -3.46 to -1.94) resulted in a significant reduction in insulin. Compared to probiotics, magnesium (-5.10 *μ*IU/mL, 95% CI: -9.32 to -0.88) and vitamin D (-3.19 *μ*IU/mL, 95% CI: -5.03 to -1.36) resulted in a greater reduction in insulin. There were no significant differences between the effectiveness of the other interventions in terms of insulin reduction.

#### 3.5.3. HOMA-IR

Table 5 shows the results of the effects of the interventions on HOMA-IR. Eleven studies reported HOMA-IR and were included in our NMA. Compared with placebo, vitamin D (-1.80, 95% CI: -2.45 to -1.16), magnesium (-1.90, 95% CI: -3.01 to -0.79), omega-3 (1.25, 95% CI: -2.00 to 0.51), zinc (-1.01, 95% CI: -1.90 to -0.12), and probiotics (-0.81, 95% CI: -1.35 to -0.28) showed a greater benefit in improving HOMA-IR. Compared to probiotics, vitamin D (-0.99, 95% CI: -1.84 to -0.14) was more effective in improving HOMA-IR. There were no significant differences between the effectiveness of the other interventions in improving HOMA-IR.

### 3.6. Rank Probabilities

**Figure 4:**
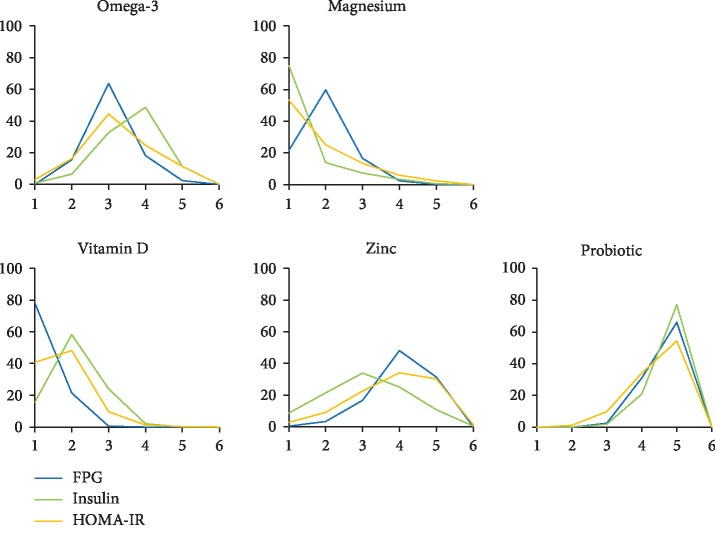
Results of different nutritional strategies ranked on the maintenance of glucose homeostasis. Lines of different colors represent different outcomes.

The NMA can estimate the best effects of each intervention on different outcomes and rank each nutritional supplementation based on SUCRA values. The larger SUCRA values indicate a better effect of intervention.  Table 6 and Figure 4 show the detailed results of the ranking. The ranking of the effectiveness of each intervention for different outcomes showed that vitamin D supplementation was the most beneficial nutritional strategy for controlling FPG and improving HOMA-IR. The most effective nutritional strategy for reducing insulin concentration was magnesium supplementation.

### 3.7. Comparison-Adjusted Funnel Plot

**Figure 5:**
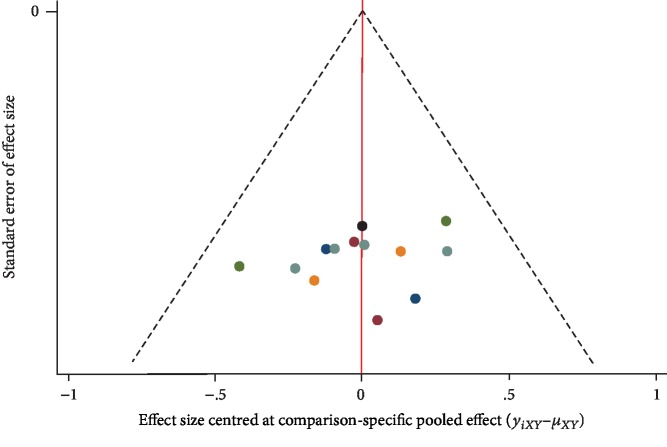
Comparison-adjusted funnel plot. Points of different colors represent different interventions.

Figure 5 shows a comparison-adjusted funnel plot. All studies on the funnel plot were symmetrically distributed on either side of the vertical line of *X* = 0, indicating that there were no significant small sample effects or publication bias.

## 4. Discussion

GDM is a special type of diabetes mellitus that can cause adverse effects in women with GDM in both the short term and long term. It is essential to control blood glucose in women with GDM. Currently, nutritional management is a widely used method, and rational nutritional strategies can not only ensure the nutritional needs of mothers and infants but also effectively control blood glucose. Most pregnant women can achieve satisfactory blood glucose levels and good pregnancy outcomes through nutrition management [35]. An exploration of the effects of different types of nutritional supplementation on glucose metabolism indicated that such supplementation can not only be used as adjunctive therapies to nutritional treatment but also appropriately meet the nutritional needs of pregnant women and fetuses. At present, studies have shown the effectiveness of vitamin D and omega-3 [36–38] in improving glucose metabolism. Direct meta-analysis studies mostly analyze the effects of supplementation with a single nutrient compared with placebo, and few studies have compared the effects of different nutritional strategies. The effect of supplementation with different nutrients on glucose metabolism in GDM patients has not been uniformly assessed. Therefore, we searched for related published RCT studies, and NMA was used to perform direct and indirect comparisons of the impact of five nutritional supplementation strategies on FPG, serum insulin, and HOMA-IR in GDM patients. The effects were quantified to identify the best nutritional supplementation strategy to provide new ideas for adjunctive therapies in GDM patients.

In our study, pairwise meta-analysis and NMA results showed that nutrient supplementation significantly decreased FPG, serum insulin, and HOMA-IR compared with the effects of the placebo. According to the Academy of Nutrition and Dietetics, nutritional therapy is the basis of GDM therapy [39], and all individuals with prediabetes and any other type of diabetes should receive individualized nutritional therapy according to the condition. The NMA results showed that vitamin D is better than omega-3, zinc, and probiotics for decreasing FPG and that magnesium is better than probiotics for decreasing FPG. Vitamin D and magnesium have certain advantages compared with probiotics for decreasing serum insulin. Vitamin D has greater benefits for improving HOMA-IR than probiotics. There were no significant differences between the other nutrients that were supplemented.

According to the results of SUCRA, vitamin D supplementation was the best for reducing FPG and improving HOMA-IR compared with the effects of the other nutritional strategies, and magnesium supplementation ranked second. Vitamin D, also known as calciferol, is mainly active in the body as 25-hydroxyvitamin D (25(OH)D), which is often used as the best indicator for measuring vitamin D levels [40]. Vitamin D deficiency is a common phenomenon after pregnancy; a study showed that at 25-28 weeks of gestation, the concentration of 25(OH)D in GDM patients is significantly reduced [41]. Appropriate vitamin D supplementation to maintain optimal 25(OH)D levels is potentially beneficial for glucose metabolism. Vitamin D deficiency is considered a potential risk factor for abnormal glucose metabolism; Zhang et al. [42] conducted a study based on data free of the Hawthorne effect, and the study indicated that low levels of vitamin D in the blood may increase the risk of GDM and that appropriate vitamin D supplementation may improve GDM status. Studies have shown that vitamin D can stimulate the body to secrete insulin under physiological conditions [43] and that it is essential for maintaining normal glucose tolerance. 25(OH)D can not only regulate insulin secretion by binding to receptors in islet *β* cells but also stimulate insulin receptor expression to promote insulin sensitivity [44], achieving the effect of decreasing blood glucose. In addition, vitamin D has antioxidant effects, which can reduce the damage to islet *β* cells and the apoptosis of islet *β* cells via active oxidative groups [45]. GDM patients can increase their concentration of 25(OH)D via vitamin D supplementation, thereby ameliorating insulin resistance and decreasing blood glucose [46].

According to the results of SUCRA, magnesium supplementation showed better results in terms of decreasing serum insulin, and in the cumulative ranking results, magnesium ranked first, followed by vitamin D. Magnesium can protect and repair islet *β* cells [47]. Deficiency of magnesium can cause changes in the structure of pancreatic cells, reduce the particles in *β* cells, and lead to insufficient insulin synthesis and secretion. Barbagallo et al. [48] showed that insufficient magnesium intake and lack of plasma magnesium can affect the process of glucose metabolism. Mg^2+^ is an important cation in cells and is a coenzyme involved in more than 300 enzymatic reactions [49]. Changes in magnesium concentration can affect islet responses, and Mg^2+^ deficiency is one of the nongenetic regulators of insulin resistance [50]. Mg^2+^ is regarded as a second messenger of insulin and plays an important role in the stability of glucose metabolism and insulin sensitivity. First, magnesium deficiency can reduce insulin receptor activity and result in insulin resistance. Second, hypomagnesemia inhibits glucose utilization in both basal and insulin-stimulated states [51]. Therefore, magnesium ions play an important role in glucose metabolism.

In summary, vitamin D and magnesium supplementation during pregnancy is more effective than supplementation with other nutrients for women with GDM. To the best of our knowledge, this is the first NMA comparing the effects of different nutritional strategies in maintaining glucose metabolic homeostasis. A particular advantage of NMA is that one can estimate the effects among different interventions by incorporating direct and indirect evidence. However, this review also has some limitations. First, we included thirteen studies; however, most of the studies included in our review are from Iran. Subjects were requested to not change their routine physical activity or usual dietary intake throughout the study; however, every country has different eating habits and routines for pregnancy care and medications that may cause differences in regulating glucose homeostasis in different countries, which may introduce uncertainty in clinical significance and influence the universality of outcomes. Moreover, some of the studies had fewer samples, and some studies had a high risk bias due to the lack of allocation concealment and blinding of outcome assessment, requiring more relevant high-quality and large-scale studies in the future. Second, the number of studies included in each intervention is limited. There are differences in the inclusion criteria for GDM and the types and doses of nutritional supplements in each study that lead to increased heterogeneity; therefore, more studies and well-controlled design are needed to decrease heterogeneity and provide more evidence. Third, because our subjects were women with GDM, the majority of the intervention durations in the studies were approximately six weeks, which may have affected the conclusion. Fourth, most of our studies were placebo-controlled trials; the number of head-to-head trials that directly compared different nutritional supplement strategies was limited, and more direct evidence of different nutritional strategies is needed to further validate our conclusions in the future.

## 5. Conclusions

Overall, vitamin D intake has a significant effect of reducing FPG and improving HOMA-IR. Magnesium intake has a superior effect of regulating serum insulin than supplementation with other nutrients. In addition, the results of our study indicate that omega-3, zinc, and probiotic supplementation are beneficial for maintaining glucose homeostasis. The present results suggest that these nutritional supplements may be considered adjunctive therapies for glycemic control in women with GDM. However, the limitations of the study may affect the clinical significance and universality of the results. Further studies are warranted to reduce the limitations of the existing evidence and to confirm the above conclusions.

## Figures and Tables

**Table 1:** Characteristics of studies included in the network meta-analysis.

Study	Country	Diagnostic criteria for gestational diabetes	Duration (weeks)	Sample size (I/C)	Mean age (years) (I/C)	Mean gestational age (weeks) (I/C)	Intervention group	Control group	Outcomes
Jamilian (2018) [22]	Iran	Based on the American Diabetes association guidelines (2014)	6	20/20	30.5/30.8	25.4/25.3	1000 mg fish oil capsules containing 180 mg EPA and 120 mg DHA twice a day	1000 mg placebo capsules twice a day	FPG, insulin, HOMA-IR
Samimi (2015) [23]	Iran	One-step: 75 g. Fasting ≥ 92 mg/dL; 1‐h ≥ 180 mg/dL; 2‐h ≥ 153 mg/dL	6	28/28	29.8/30.3	25.7/25.4	1000 mg omega-3 capsules containing 180 mg EPA and 120 mg DHA per day	1000 mg placebo capsules containing 500 mg liquid paraffin per day	FPG, insulin, HOMA-IR
Jamilian (2017) [24]	Iran	One-step: 75 g. FPG ≥ 92 mg/dL; 1‐h ≥ 180 mg/dL; 2‐h ≥ 153 mg/dL	6	35/35	30.7/31.5	24–28	1000 mg omega-3 containing 360 mg EPA and 240 mg DHA twice a day+vitamin D placebo	50,000 IU vitamin D every 2 weeks+omega-3 placebo	FPG, insulin, HOMA-IR
Asemi (2015) [25]	Iran	One-step: 75 g. Fasting ≥ 92 mg/dL: 1‐h ≥ 180 mg/dL; or 2‐h ≥ 153 mg/dL	6	35/35	29.1/29.4	24–28	250 mg magnesium oxide tablets per day	250 mg placebo tablets per day	FPG, insulin, HOMA-IR
Jamilian (2017) [26]	Iran	FPG < 105 mg/dL; blood sugar 2-hour post − prandial < 120 mg/dL	6	20/20	27.8/27.1	N/S	250 mg magnesium oxide per day	250 mg placebo per day	FPG
Asemi (2013) [27]	Iran	Two steps: First step: 50 g. 1-h plasma glucose concentrations > 140 mg/dL Second step: 100 g. Fasting > 95 mg/dL; 1‐h ≥ 180 mg/dL; 2‐h ≥ 155 mg/dL; 3‐h ≥ 140 mg/dL	6	27/27	31.7/31.8	24–28	Capsules containing 50,000 IU vitamin D_3_ 2 times during the study	Placebo capsules 2 times during the study	FPG, insulin, HOMA-IR
Li (2016) [28]	China	Fasting plasma glucose > 92 mg/dL; 1‐h ≥ 180 mg/dL; 2‐h ≥ 153 mg/dL	16	48/49	29.0/28.3	14.5/14.2	100 g vitamin D yogurt drink containing 500 IU vitamin D3 twice a day	100 g plain yogurt drink without any vitamin D3 supplement twice a day	FPG, insulin, HOMA-IR
Karamali (2015) [30]	Iran	One-step: 75 g. Fasting ≥ 92 mg/dL; 1‐h ≥ 180 mg/dL; or 2‐h ≥ 153 mg/dL	6	29/29	29.9/29.4	24–28	233 mg zinc gluconate tablets containing 30 mg zinc per day	233 mg placebo tablets (starch) per day	FPG, insulin, HOMA-IR
Roshanravan (2015) [30]	Iran	Two-steps: First step: 50 g. 1-h blood sugar ≥ 130 mg/dL Second step: 75 g. FBS > 92 mg/dL; 1‐h ≥ 180 mg/dL; 2‐h ≥ 153 mg/dL	8	22/22	29.5/29.8	24–28	30 mg zinc gluconate tablets per day between meals	30 mg placebo tablets (starch) per day between meals	FPG, insulin, HOMA-IR
Babadi (2018) [31]	Iran	Diagnosed with GDM by a “one-step” based on the American Diabetes Association guidelines (2014)	6	24/24	28.8/29.0	24–28	A probiotic capsule containing *Lactobacillus acidophilus*, *Lactobacillus casei*, *Bifidobacterium bifidum*, and *Lactobacillus fermentum* (2 × 10^9^ CFU/g each) per day	A placebo (corn starch) per day	FPG, insulin, HOMA-IR
Badehnoosh (2018) [32]	Iran	One-step: 75 g. FPG ≥ 92 mg/dL; 1‐h ≥ 180 mg/dL; 2‐h ≥ 153 mg/dL	6	30/30	28.8/27.8	25.7/25.6	A probiotic capsule containing *Lactobacillus acidophilus*, *Lactobacillus casei*, and *Bifidobacterium bifidum* (2 × 10^9^ CFU/g each) strains per day	A placebo capsule (starch) per day	FPG
Karamali (2016) [33]	Iran	One-step: 75 g. FPG ≥ 92 mg/dL; 1‐h ≥ 180 mg/dL; 2‐h ≥ 153 mg/dL	6	30/30	31.8/29.7	24–28	Probiotic capsules containing *L*. *acidophilus*, *L*. *casei*, and *B*. *bifidum* (2 × 10^9^ CFU/g each) strains per day	Placebo capsules (starch) per day	FPG, insulin, HOMA-IR
Kijmanawat (2019) [34]	Thailand	One-step: 75 g. FPG ≥ 92 mg/dL; 1‐h ≥ 180 mg/dL; 2‐h ≥ 153 mg/dL	4	28/29	32.5/30.7	27.3/28.0	Probiotic capsule containing *Lactobacillus acidophilus* and *Bifidobacterium bifidum* (1 × 10^9^ CFU/g each) per day	Placebo capsule (gelatin) per day	FPG, insulin, HOMA-IR

I/C: intervention group/control group; N/S: not stated; h: hours; mg/dL: milligrams per deciliter; mmol/L: millimoles per liter; CFU/g: colony-forming units per gram; FPG: fasting plasma glucose; HOMA-IR: homeostasis model assessment-insulin resistance; EPA: eicosapentaenoic acid; DHA: docosahexaenoic acid.

**Table 2:** Results of pairwise meta-analysis.

	Studies	Patients	WMD (95% CI)	*I* ^2^
FPG				
Placebo vs. omega-3	2	96	-5.93 (-10.29, -1.57)	0%
Placebo vs. magnesium	2	110	-10.59 (-13.68, -7.50)	0%
Placebo vs. vitamin D	2	151	-13.17 (-15.95, -10.39)	0%
Placebo vs. zinc	2	102	-6.42 (-10.18, -2.65)	0%
Placebo vs. probiotic	4	225	-5.49 (-8.05, -2.93)	25%
Insulin				
Placebo vs. omega-3	2	96	-3.22 (-6.21, -0.24)	28%
Placebo vs. vitamin D	2	151	-6.23 (-8.05, -4.40)	29%
Placebo vs. zinc	2	102	-4.61 (-7.04, -2.18)	62%
Placebo vs. probiotic	3	165	-2.70 (-3.46, -1.94)	0%
HOMA-IR				
Placebo vs. omega-3	2	96	-1.01 (-1.81, -0.21)	17%
Placebo vs. vitamin D	2	151	-1.97 (-2.51, -1.42)	5%
Placebo vs. zinc	2	102	-0.97 (-1.70, -0.23)	81%
Placebo vs. probiotic	3	165	-0.69 (-0.88, -0.50)	33%

**Table 3:** Results of the network meta-analysis on FPG.

Magnesium	2.10 (-2.07, 6.26)	**10.59 (7.50, 13.68)**	**5.22 (1.44, 9.00)**	-1.54 (-5.47, 2.39)	4.17 (-0.70, 9.04)
-2.10 (-6.26, 2.07)	Omega-3	**8.49 (5.70, 11.28)**	3.12 (-0.42, 6.66)	**-3.64 (-5.77, -1.51)**	2.07 (-2.61, 6.76)
**-10.59 (-13.68, -7.50)**	**-8.49 (-11.28, -5.70)**	Placebo	**-5.37 (-7.54, -3.19)**	**-12.13 (-14.55, -9.70)**	**-6.42 (-10.18, -2.65)**
**-5.22 (-9.00, -1.44)**	-3.12 (-6.66, 0.42)	**5.37 (3.19, 7.54)**	Probiotics	**-6.76 (-10.02, -3.50)**	-1.05 (-5.40, 3.30)
1.54 (-2.39, 5.47)	**3.64 (1.51, 5.77)**	**12.13 (9.70, 14.55)**	**6.76 (3.50, 10.02)**	Vitamin D	**5.71 (1.23, 10.19)**
-4.17 (-9.04, 0.70)	-2.07 (-6.76, 2.61)	**6.42 (2.65, 10.18)**	1.05 (-3.30, 5.40)	**-5.71 (-10.19, -1.23)**	Zinc

Comparing the effects (weighted mean differences: WMDs) of all nutritional strategies and 95% confidence intervals (95% CIs). Significant results are shown in bold.

**Table 4:** Results of the network meta-analysis on insulin.

Magnesium	3.68 (-1.05, 8.41)	**7.80 (3.65, 11.95)**	**5.10 (0.88, 9.32)**	1.91 (-2.57, 6.38)	3.02 (-2.33, 8.36)
-3.68 (-8.41, 1.05)	Omega-3	**4.12 (1.86, 6.38)**	1.42 (-0.97, 3.81)	-1.77 (-4.03, 0.48)	-0.66 (-4.73, 3.40)
**-7.80 (-11.95, -3.65)**	**-4.12 (-6.38, -1.86)**	Placebo	**-2.70 (-3.46, -1.94)**	**-5.89 (-7.57, -4.22)**	**-4.78 (-8.16, -1.41)**
**-5.10 (-9.32, -0.88)**	-1.42 (-3.81, 0.97)	**2.70 (1.94, 3.46)**	Probiotics	**-3.19 (-5.03, -1.36)**	-2.08 (-5.55, 1.38)
-1.91 (-6.38, 2.57)	1.77 (-0.48, 4.03)	**5.89 (4.22, 7.57)**	**3.19 (1.36, 5.03)**	Vitamin D	1.11 (-2.66, 4.88)
-3.02 (-8.36, 2.33)	0.66 (-3.40, 4.73)	**4.78 (1.41, 8.16)**	2.08 (-1.38, 5.55)	-1.11 (-4.88, 2.66)	Zinc

Comparing the effects (weighted mean differences: WMDs) of all nutritional strategies and 95% confidence intervals (95% CIs). Significant results are shown in bold.

**Table 5:** Results of the network meta-analysis on HOMA-IR.

Magnesium	0.65 (-0.69, 1.98)	**1.90 (0.79, 3.01)**	1.09 (-0.14, 2.32)	0.10 (-1.19, 1.38)	0.89 (-0.53, 2.31)
-0.65 (-1.98, 0.69)	Omega-3	**1.25 (0.51, 2.00)**	0.44 (-0.50, 1.39)	-0.55 (-1.30, 0.21)	0.25 (-0.93, 1.42)
**-1.90 (-3.01, -0.79)**	**-1.25 (-2.00, -0.51)**	Placebo	**-0.81 (-1.35, -0.28)**	**-1.80 (-2.45, -1.16)**	**-1.01 (-1.90, -0.12)**
-1.09 (-2.32, 0.14)	-0.44 (-1.39, 0.50)	**0.81 (0.28, 1.35)**	Probiotics	**-0.99 (-1.84, -0.14)**	-0.20 (-1.21, 0.82)
-0.10 (-1.38, 1.19)	0.55 (-0.21, 1.30)	**1.80 (1.16, 2.45)**	**0.99 (0.14, 1.84)**	Vitamin D	0.79 (-0.31, 1.90)
-0.89 (-2.31, 0.53)	-0.25 (-1.42, 0.93)	**1.01 (0.12, 1.90)**	0.20 (-0.82, 1.21)	-0.79 (-1.90, 0.31)	Zinc

Comparing the effects (weighted mean differences: WMDs) of all nutritional strategies and 95% confidence intervals (95% CIs). Significant results are shown in bold.

**Table 6:** Ranking results of the comparative effects of different nutritional strategies on the maintenance of glucose homeostasis.

Treatments	FPG		Insulin		HOMA-IR	
	SUCRA (%)	Mean rank	SUCRA (%)	Mean rank	SUCRA (%)	Mean rank
Placebo	0	6.0	0.1	6.0	0.3	6.0
Omega-3	58.5	3.1	47.3	3.6	55.0	3.3
Magnesium	80.0	2.0	92.0	1.4	84.2	1.8
Vitamin D	95.5	1.2	77.5	2.1	85.5	1.7
Zinc	38.6	4.1	58.2	3.1	43.3	3.8
Probiotics	27.3	4.6	25.0	4.8	31.6	4.4

FPG: fasting plasma glucose; HOMA-IR: homeostasis model assessment-insulin resistance.

## Supplementary Materials

Supplementary Materials Annex 1: detailed search strategy.
