# Supplementary material for: Effects of Nutritional Strategies on Glucose Homeostasis in Gestational Diabetes Mellitus: A Systematic Review and Network Meta-Analysis
Source: J Diabetes Res. 2020 Feb 23;2020:6062478. doi: 10.1155/2020/6062478 (PMC7060856; doi:10.1155/2020/6062478)
Supplement: Supplementary Materials — Annex 1: detailed search strategy. [file 6062478.f1.pdf]

Annex 1: Detailed search strategy

PubMed

#1 "Diabetes, Gestational"[Mesh] 11560

#2 Pregnancy-Induced Diabetes[Title/Abstract] 12

#3 Gestational Diabetes Mellitus[Title/Abstract] 7574

#4 GDM[Title/Abstract] 6581

#5 #1OR#2OR#3OR#4 14283

#6 nutrient*[Title/Abstract] 133504

#7 nutrition[Title/Abstract]) 164036

#8 dietary supplement[Title/Abstract]) 5066

#9 protein[Title/Abstract]) 2391555

#10 amino acids[Title/Abstract]) 206866

#11 fatty acids[Title/Abstract]) 132223

#12 vitamin[Title/Abstract]) 192088

#13 mineral[Title/Abstract]) 105366

#14 antioxidant[Title/Abstract]) 163415

#15 phytochemical[Title/Abstract] 12190

#16 #6OR#7OR#8OR#9OR#10OR#11OR#12OR#13OR#14OR#15 3188131

#17 "Randomized Controlled Trial" [Publication Type] 489079

#18 controlled trial[Title/Abstract]) 119968

#19 clinical trial[Title/Abstract]) 134527

#20 random*[Title/Abstract]) 1066592

#21 RCT[Title/Abstract] 20238

#22 #17OR#18OR#19OR#20OR#21 1257451

#23 #5 AND #16 AND #22 226

Cochrane

#1 MeSH descriptor:[diabetes, gestational] explode all trees 795

#2 pregnancy-induced diabetes :ab,ti,kw 123 in trials to July 2019

#3 gestational diabetes mellitus :ab,ti,kw 2068 in trials to July 2019

#4 GDM:ab,ti,kw 1101 in trials to July 2019

#5 #1OR#2OR#3OR#4 2348 in trials to July 2019

#6 nutrient*:ab,ti,kw 5970 in trials to July 2019

#7 nutrition:ab,ti,kw 21828 in trials to July 2019

#8 dietary supplement:ab,ti,kw 5555 in trials to July 2019

#9 protein:ab,ti,kw 68303 in trials to July 2019

#10 amino acids:ab,ti,kw 4550 in trials to July 2019

#11 fatty acids:ab,ti,kw 12410 in trials to July 2019

#12 vitamin:ab,ti,kw 25972 in trials to July 2019

#13 mineral:ab,ti,kw 11454 in trials to July 2019

#14 antioxidant:ab,ti,kw 9418 in trials to July 2019

#15 phytochemical:ab,ti,kw 147 in trials to July 2019

#16 #6OR#7OR#8OR#9OR#10OR#11OR#12OR#13OR#14OR#15 135166 in trials to July 2019

#17   #5AND#16   513 in trials

EMbases

#1 diabetes, gestational /exp   33396

#2 pregnancy-induced diabetes :ab,ti 14

#3 gestational diabetes mellitus :ab,ti 10328

#4 GDM:ab,ti   10641

#5 #1OR#2OR#3OR#4   34605

#6 nutrient*:ab,ti   155386

#7 nutrition:ab,ti   195429

#8 dietary supplement:ab,ti   6344

#9 protein:ab,ti   2800622

#10 amino acids:ab,ti   209001

#11 fatty acids:ab,ti   148986

#12 vitamin:ab,ti   229764

#13 mineral:ab,ti   132889

#14 antioxidant:ab,ti   209001

#15 phytochemical:ab,ti   24172

#16#6OR#7OR#8OR#9OR#10OR#11OR#12OR#13OR#14OR#15   3634797

#17 controlled trial:ab,ti   153510

#18 clinical trial:ab,ti   185807

#19 random*:ab,ti   1420533

#20 RCT:ab,ti 33205

#21 #17OR#18OR#19OR#20 1535946

#22 #5 AND #16 AND #21 382
